# Modified aptamers as reagents to characterize recombinant human erythropoietin products

**DOI:** 10.1038/s41598-020-75713-2

**Published:** 2020-10-29

**Authors:** Wojciech Jankowski, H. A. Daniel Lagassé, William C. Chang, Joseph McGill, Katarzyna I. Jankowska, Amy D. Gelinas, Nebojsa Janjic, Zuben E. Sauna

**Affiliations:** 1 Hemostasis Branch, Division of Plasma Protein Therapeutics, Center for Biologics Evaluation and Research, Food and Drug Administration, Silver Spring, MD USA; 2 Laboratory of Cellular Hematology, Division of Blood Components and Devices, Center for Biologics Evaluation and Research, Food and Drug Administration, Silver Spring, MD USA; 3 SomaLogic, Inc., Boulder, CO USA

**Keywords:** Biophysics, Biochemical assays, Biophysical chemistry, Diagnostics, High-throughput screening, Chemical modification

## Abstract

Reliable and reproducible monitoring of the conformational state of therapeutic protein products remains an unmet technological need. This need is amplified by the increasing number of biosimilars entering the drug development pipeline as many branded biologics are reaching the end of their market exclusivity period. Availability of methods to better characterize protein conformation may improve detection of counterfit and unlicensed therapeutic proteins. In this study, we report the use of a set of modified DNA aptamers with enhanced chemical diversity to probe the conformational state of 12 recombinant human erythropoietin (rHuEPO) therapeutic protein products; one FDA-licensed rHuEPO originator biological product, three rHuEPO products that are approved for marketing in the US or EU as biosimilars, and eight rHuEPO products that are not approved for marketing in the US or EU. We show that several of these modified aptamers are able to distinguish rHuEPO reference products or approved biosimilars from non-licensed rHuEPO products on the basis of differences in binding kinetics and equilibrium affinity constants. These reagents exhibit sensitivity to the conformational integrity of various forms of rHuEPO and as such represent powerful, simple-to-use analytical tools to monitor the conformational integrity of therapeutic-proteins during manufacture and to screen for and identify both substandard and counterfeit products.

## Introduction

While the methods to assess protein sequence and even secondary configuration are robust, characterization of higher-order (tertiary and quaternary) structures of proteins has lagged. Thus, the development of robust analytical methods to routinely and inexpensively determine differences in higher-order structures of proteins would enhance comparability and biosimilarity assessments. In a previous study we demonstrated that a panel of aptamers probing different regions of a protein can be used to detect subtle differences in protein conformation^1^. More recently, six aptamers specific to the therapeutic antibody rituximab were used to compare a non-US licensed reference product approved in the EU, a non-US licensed biosimilar approved in the EU and a non-US licensed rituximab product not licensed in the EU^2^. While the aptamers did not demonstrate differences between the European biosimilar and its reference product, they detected conformational differences between the reference product/biosimilar and the product marketed in India suggesting that this technology may be useful in comparability and biosimilarity exercises. Aptamers have also been described as novel detection^3^ or targeting agents^4^, and notably used in food safety analysis to detect biotoxins and other harmful substances^5,6^.

In this study we have refined the methodology of using aptamers to detect conformational changes and provide a proof-of-principle application of the methodology to recombinant human erythropoietin (rHuEPO). For this purpose, we have generated four next-generation modified aptamers called SOMAmer (Slow Off-rate Modified Aptamer) reagents that have different sequences and compositions, meaning they bind to their protein target with non-identical interaction surfaces. We used these reagents in a high-throughput analysis of binding parameters (association and dissociation rate constants, and equilibrium dissociation constants) using Bio-Layer Interferometry (BLI). The method was used to comprehensively characterize conformational differences between an FDA-licensed rHuEPO originator biological product (hereafter “Epogen/Procrit”), three rHuEPO products that are approved for marketing in the US or EU as biosimilars (for purposes of this study, these US and non US-licensed rHuEPO biosimilar products will be referred to as “US and non-US-licensed rHuEPO biosimilar products (BiSi)”), and eight rHuEPO products that are not approved for marketing in the US or EU [for purposes of this study, these products will be referred to as “non-licensed rHuEPO products (NLP)”]. There were minimal differences detected between Epogen/Procrit and the US and non-US-licensed rHuEPO biosimilar products. However, we detected significant disparities between these products and the non-licensed products.

## Results

### Preparation and characterization of SOMAmers

**Figure 1:**
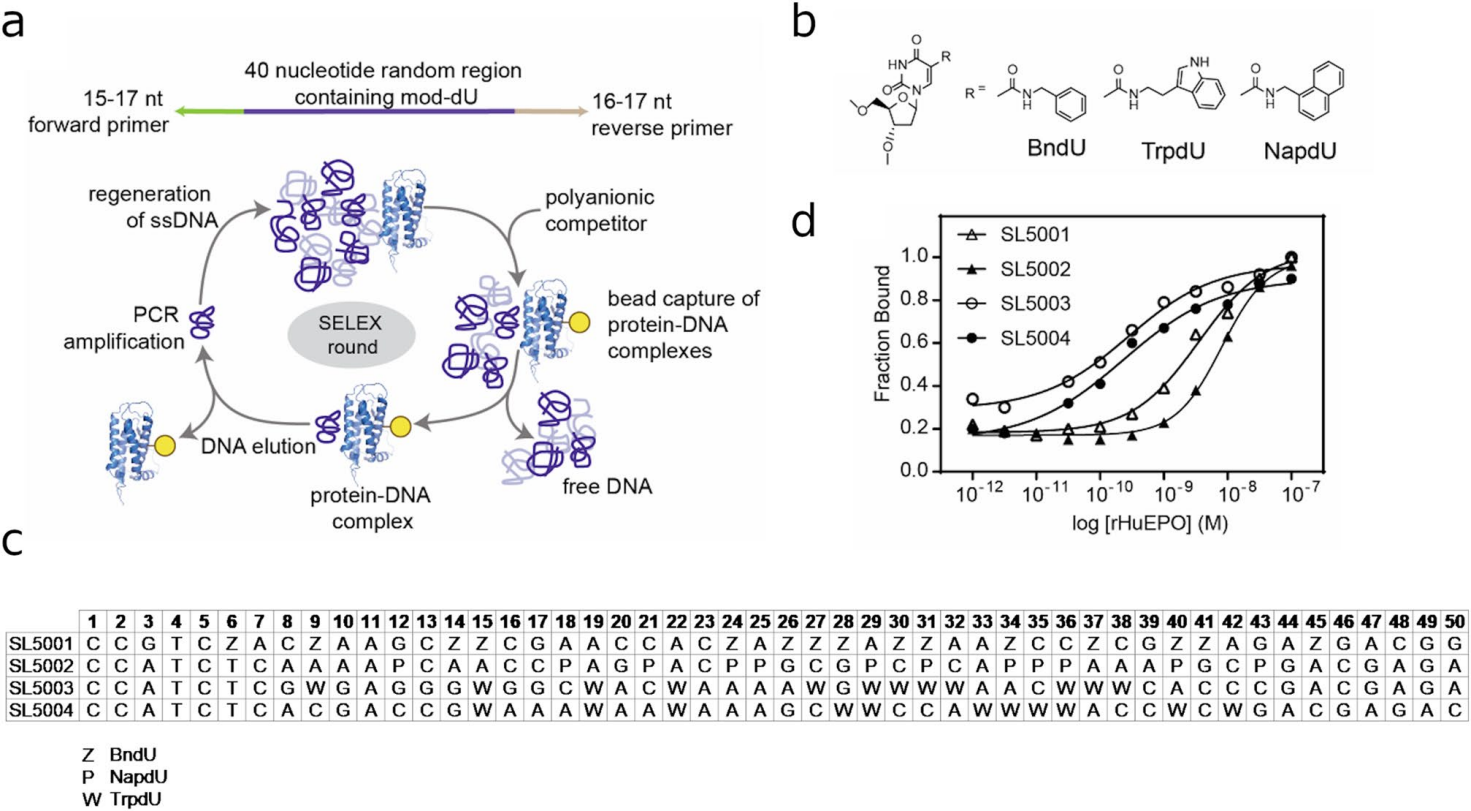
Preparation and characterization of SOMAmers. (**a**) Illustration of SELEX approach to identify anti-rHuEPO SOMAmers (PDB ID: 1EER^38^); (**b**) Structures of modified nucleosides used to generate the anti-rHuEPO SOMAmers. (**c**) Sequences of SOMAmer reagents used in this work. (**d**) Dose–response curve to determine affinities of anti-rHuEPO SOMAmers for rHuEPO using a filter-binding assay.

Modified aptamers targeting rHuEPO were discoverved by the systematic evolution of ligands by exponential enrichment (SELEX) process^5^ with conditions that favored slow dissociation rates (Fig. 1a). Three modified nucleotide libraries with a modified 40 nucleotide random region each uniformly substituted at all dT positions with either (1) 5-(*N*-benzylcarboxamide)-2′-deoxyuridine (BndU), (2) 5-napthylmethylaminocarbonyl 2′-deoxyuridine (NapdU) or (3) 5-tryptaminocarbonyl-2′deoxyuridine (TrpdU) were used in SELEX (Fig. 1b). Following the completion of SELEX, sequences representing a high percentage of the affinity-enriched pools (SL5001, 2%; SL5002, 29%; SL5003 3%; SL5004, 5%) were synthesized as 50-mers (40 N random region plus 5 nucleotides from the 5′ and 3′ primer regions) for affinity screening with rHuEPO. Four of the aptamers tested (one each from BndU and NapdU libraries and two from TrpdU library, Fig. 1c) with affinities below 20 nM (Fig. 1d, Supplementary Table S1) were selected for further rHuEPO characterization studies and were thus synthesized with a 5′ biotin for use in BLI.

### Different versions of erythropoietin used in the study

**Figure 2:**
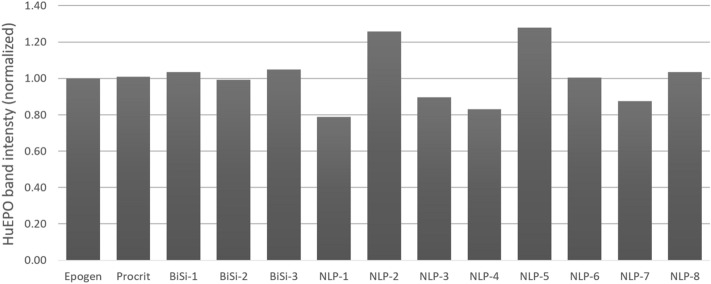
Quantification of the rHuEPO immunoblot. The different versions of rHuEPO used in the study were subjected to SDS-PAGE followed by immuno-blotting using a rabbit polyclonal IgG specific to human EPO (Supplementary Fig. S1). The relative intensity of rHuEPO bands is normalized to Epogen/Procrit.

Several recent studies have reported comparisons of biochemical and biophysical properties of recombinant human erythropoietin (rHuEPO) products^6,7^. In addition, several biosimilar versions of the rHuEPO have been approved by regulatory agencies in the US and Europe^8–13^. Here we used the panel of four anti-rHuEPO SOMAmer reagents described above to compare three classes of rHuEPO: (1) Epogen/Procrit; (2) US and non-US-licensed rHuEPO biosimilar products (BiSi-1, BiSi-2, BiSi-3); and (3) non-licensed rHuEPO products (NLP-1 to NLP-8). We and others have previously used aptamers that probe different regions of a protein to detect subtle differences in protein conformation^1,2^. We adapted this method to compare the three groups of rHuEPO products described above. Reducing SDS-PAGE analysis followed by immuno-blotting showed each of the rHuEPO products tested contained product band ~ 37 kDa (Supplementary Fig. S1). No significant truncated isoforms or product aggregates were observed by immunoblot for the rHuEPO products (Supplementary Fig. S1). However, quantification of the ~ 37 kDa band on an immunoblot showed inconsistency in the band intensities for NLP 1–5 and NLP-7 (Fig. 2).

### Using biolayer interferometry (BLI) to measure kinetic parameters of modified aptamer-rHuEPO interactions

**Figure 3:**
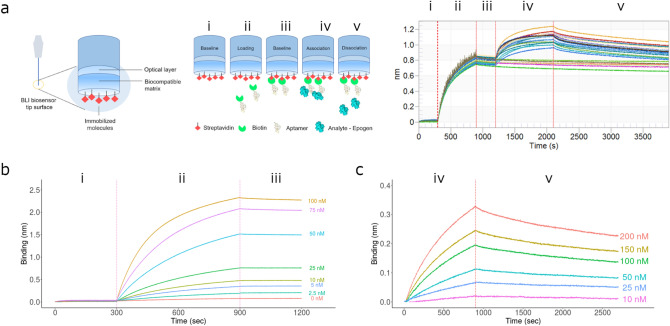
Using BLI to characterize the binding kinetics of the anti-rHuEPO SOMAmers to rHuEPO. (**a**) Illustation (left) and real-time sensorgram traces (right) of the BLI protocol that uses streptavidin-coated biosensors (i) which were loaded with biotinylated SOMAmer (ii). In subsequent steps, following a running buffer baseline step (iii), the association (iv) and dissociation (v) of rHuEPO products to immobilized SOMAmers were monitored in real time. (**b**) Representative real-time BLI sensorgram traces monitoring the optimization of SOMAmer (SL5002) loading over a concentration range (100–2.5 nM): (i) baseline; (ii) loading/binding of biotinylated SOMAmer to streptavidin-coated sensors; (iii) washing of excesss SOMAmer and equilibration in running buffer. (**c**) Representative real-time BLI sensorgram traces monitoring the optimization of rHuEPO (Epogen/Procrit) concentrations (200–10 nM) used in the assay following loading of SOMAmer (50 nM SL5002) to streptavidin-coated biosensors: (iv) association of Epogen/Procrit with SOMAmer SL5002; (v) dissociation of the Epogen/Procrit:SOMAmer complex.

Several label-free technologies are available to determine biomolecular binding kinetics parameters such as association rate constant (k_a_), dissociation rate constant (k_d_), and equilibrium dissociation constant (K_D_). We selected BLI as the method of choice for determining kinetic constants since it offers a high-throughput platform for determining the rate constants for SOMAmer-rHuEPO interactions. A typical experiment (Fig. 3a) to determine SOMAmer-rHuEPO binding constants involves (1) a pre-loading baseline reading; (2) loading/binding of the biotinylated SOMAmer reagent to the streptavidin-coated sensor; (3) a pre-binding baseline reading; (4) association of rHuEPO with the SOMAmer; (5) SOMAmer-rHuEPO dissociation. Optimization of the BLI experimental conditions involved titration of the SOMAmer reagent (Fig. 3b) and rHuEPO (Fig. 3c) to ensure sufficient signal:noise ratio, 1:1 binding kinetics, and accurate measurement of the association and dissociation rate constants for low nanomolar affinity interactions. The k_a_ and k_d_ values were obtained using the Octet System Data Analysis software (Fig. 3c).

### Specificity of SOMAmer reagents to rHuEPO

**Figure 4:**
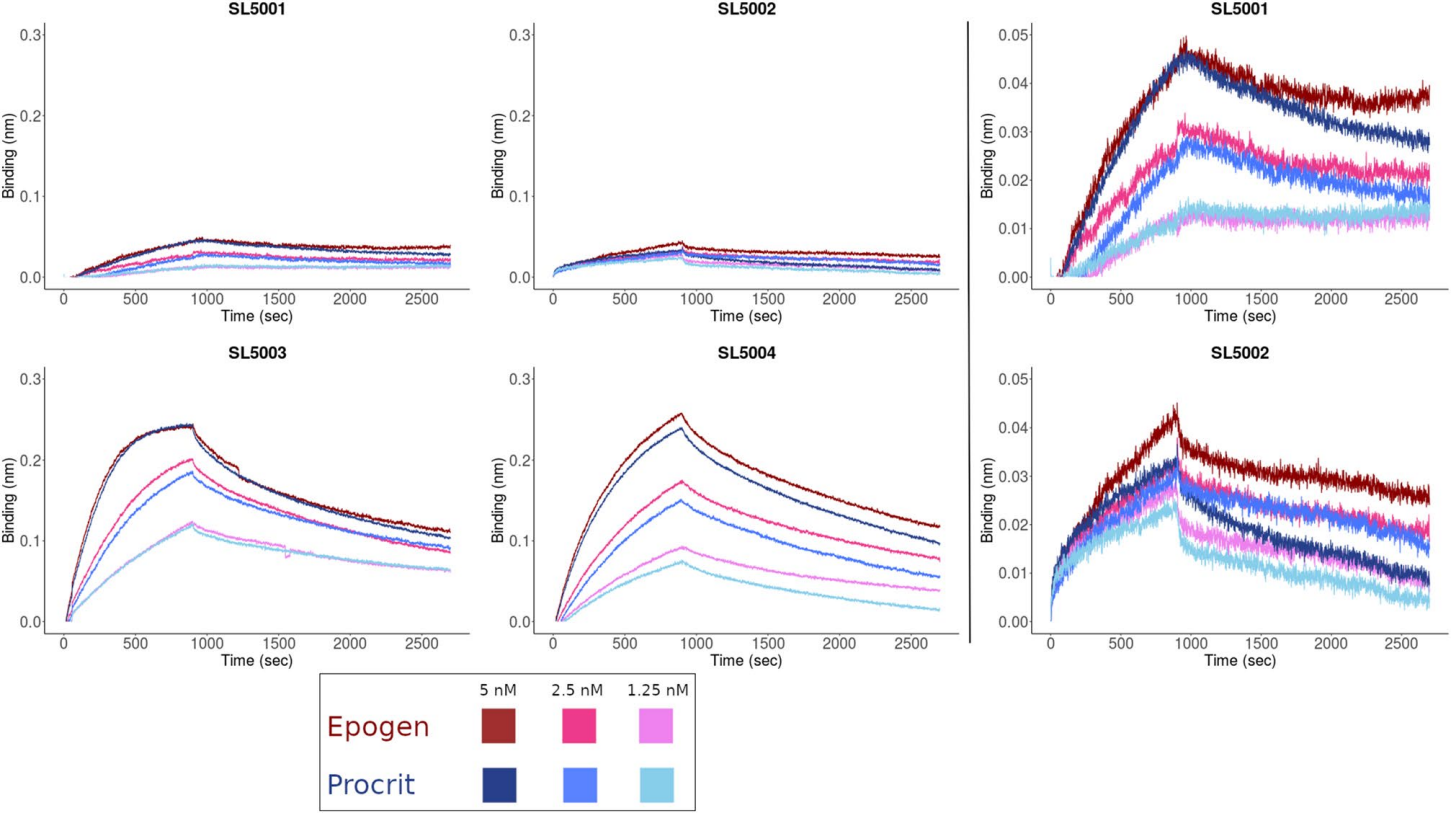
Association and dissociation constants for SOMAmer (SL5001, SL55002, SL5003 and SL55004) binding to rHuEPO (Epogen and Procrit). BLI sensorgrams depicting three concentrations of Epogen or Procrit (1.25 nM, 2.5 nM and 5 nM) used to calculate the association (k_a_) and dissociation (k_d_) constants for the 4 SOMAmers used in this study. The SOMAmers SL5001 and SL5002 exhibit lower binding to rHuEPO, thus the sensorgrams on the right use a different scale to better visualize the binding kinetics. All binding kinetic traces were analyzed using a 1:1 binding model by the Octet System Data Analysis software version 8.2 and exhibited goodness-of-fit values (R^2^) > 0.95.

We determined the binding kinetics of the four SOMAmer reagents to Epogen/Procrit using BLI (Supplementary Table S2). Each of the four SOMAmers bind with low nanomolar affinity, however, overall we observed differences in the amplitude of the binding signal generated and the association and dissociation rate constants (Fig. 4). The relative ranking of the binding affinity for each of the SOMAmer reagents for Epogen/Procrit as measured by BLI were comparable with the affinity measurements generated by filter-binding assay (Fig. 1c).

**Figure 5:**
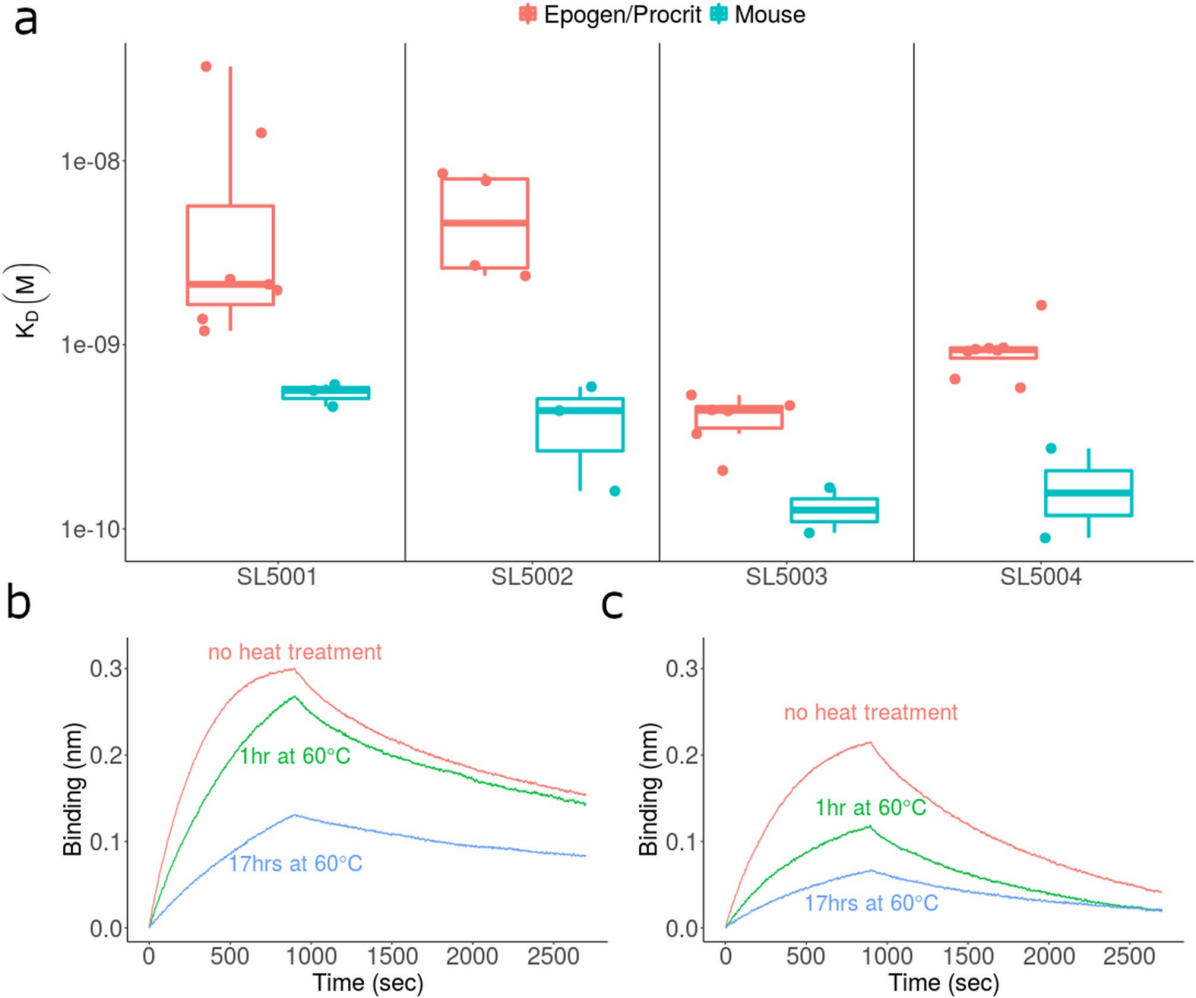
The anti-rHuEPO SOMAmers bind specifically to rHuEPO and are sensitive to the conformation of the protein. (**a**) Comparision of binding affinity (K_D_) of SOMAmers to recombinant human and murine erythropoietins (rHuEPO and rMuEPO respectively) as measured by BLI. (**b**,**c**) BLI sensorgram traces for Epogen/Procrit association and dissociation to SOMAmers SL5003 (**b**) and SL5004 (**c**). For thermal stability studies, SOMAmer:rHuEPO binding was measured following incubation of Epogen/Procrit at 60 °C for 0 h (red), 1 h (green) or 17 h (blue).

Using the BLI methodology standardized in the previous section, we estimated the binding kinetics of rHuEPO and recombinant murine EPO (rMuEPO). Although the four modified aptamers used in this study were selected for binding to rHuEPO, we assessed their ability to bind to rMuEPO, which has 78% amino acid sequence identity with rHuEPO. The binding affinities are 6.43 (95% CI 3.66–11.31, p = 1.83 × 10^–7^)-fold higher for rMuEPO compared to the affinities for rHuEPO while controlling for the variation due to SOMAmer (Fig. 5a). Additionally, we observed dramatically compromized binding to rHuEPO that has been thermally denatured upon incubation at 60 °C (Fig. 5b,c). These results suggest that the modified aptamers are specific to rHuEPO conformation and are sensitive to changes that result from heat treatment.

### Comparing the binding of SOMAmer reagents to different rHuEPO products

**Figure 6:**
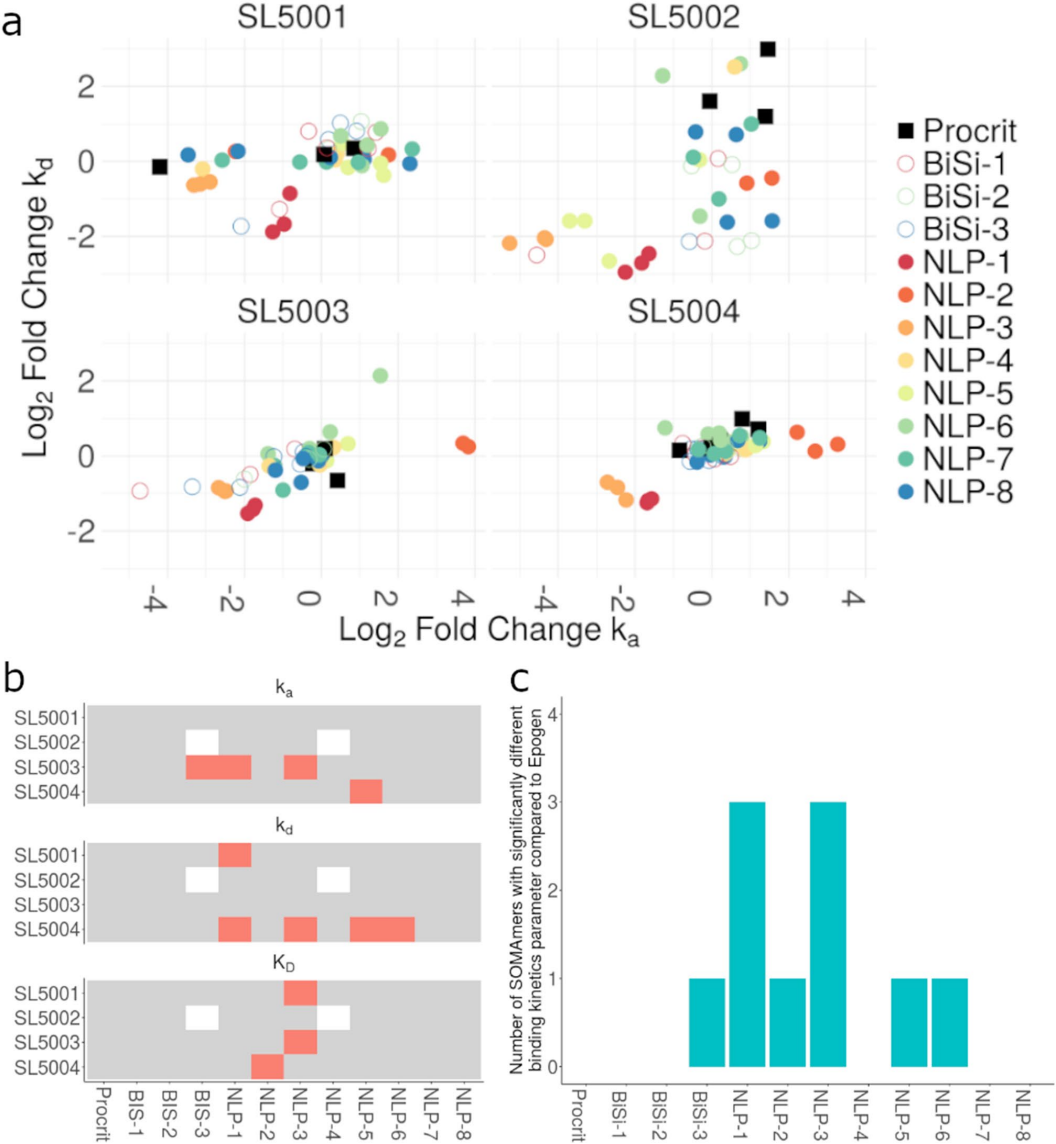
Binding kinetics of anti-rHuEPO SOMAmers to all rHuEPO products. (**a**) Fold change in the kinetics (k_a_ and k_d_) of SOMAmer-rHuEPO binding. The fold-change represents the k_a_ (x-axis) and k_d_ (y-axis) values for individual rHuEPO products (shown on the graph compared to the corresponding values for the reference product, Epogen). (**b**) All rHuEPO products tested in this study are shown on the x-axis. For each of the four SOMAmers, if the relevant measurement (k_a_, top; k_d_, middle; K_D_, bottom) was significantly different between Epogen and the rHuEPO product (based on a t test), the SOMAmer-rHuEPO combination is depicted in red. SOMAmer-rHuEPO combinations with insufficient data to perform a t test are depicted in white. (**c**) For each of the rHuEPO products, the number of SOMAmers showing a significantly different binding kinetics parameter (k_a_, k_d_, or K_D_) compared to the reference product, Epogen, are depicted. Testing Epogen/Procrit against itself, the value is, as expected, 0. The rHuEPO products with a value above 0 may be considered conformationally different from the reference product.

There are several rHuEPO products marketed throughout the world. The rHuEPO products legally marketed in the US and EU are either the reference product or biosimilars. There are numerous other rHuEPO products sold predominantly in Asia that are not licensed in the US or EU. We compared the binding kinetics of all three classes of rHuEPO products using BLI. Figure 6a compares the k_a_ and k_d_ values for the binding of the four SOMAmers to each of the rHuEPO products surveyed in this study. We have plotted the Log_2_ fold change compared to Epogen. In general these results suggest that the affinities of the non-licensed rHuEPO products show a greater deviation. To simplify the presentation we have represented the k_a_, k_d_ and K_D_ values that were significantly different (Fig. 6b, Supplementary Table S3). In Fig. 6b we observe that no SOMAmer detects significant changes in the binding kinetics between different lots of Epogen/Procrit. Based on this criterion, we observed many (though not all) non-licensed rHuEPO products have significantly different binding kinetics to one or more SOMAmers (Fig. 6c). On the other hand, Epogen/Procrit, and the US and non-US licensed rHuEPO biosimilar products have similar binding kinetics.

## Discussion

The last 2 decades have seen a major expansion in the use of recombinant proteins as therapeutics. These medicines now treat diverse diseases and are part of routine medical practice. Notwithstanding the fact that the manufacture and licensure of this class of molecules is more challenging than small molecule drugs, they have proven extremely successful due to their targeted efficacy (e.g. monoclonal antibodies) and limited side effects^16^. Additionally, proteins can provide complex biological functions, such as enzyme activity, which cannot be replicated with small molecules^17^. Reliably and reproducibly monitoring the higher-order structure of therapeutic proteins remains an unmet technological need. These techniques are necessary during the routine manufacture of therapeutic proteins. A key element in the development and licensure of a biosimilar is a comparative analytical assessment of the proposed biosimilar and the reference product^16–21^.

We have previously described the advantage of using aptamers to rapidly, routinely and reliably screen therapeutic proteins for changes or differences in higher-order structure^1^. Subsequently, the method has been applied in several specific instances^2,22^. Here we describe the use of SOMAmer reagents for comparing the conformational differences between different rHuEPO products. Compared to traditional aptamers, modified aptamers are able to access a wider range of chemically diverse epitopes due to their unique protein-like side chains^23,24^. Unmodified aptamers predominantly bind electropositive patches on proteins, mainly utilizing hydrogen bonds and charge-charge interactions^25^. In contrast, the hydrophobic moieties on modified aptamers provide increased opportunities for interactions with hydrophobic surfaces generally not receptive to conventional aptamers. The larger and more diverse surface accessibility of modified aptamers means combinations of reagents are able to probe multiple regions of the target, thus providing a comprehensive analysis of potential structural variances. The anti-rHuEPO SOMAmer reagents described in this study demonstrate an alternative approach to generate high affinity anti-rHuEPO aptamers^30^ and contain distinct nucelotide sequences from previously identified aptamers. He et al., further optimized their anti-rHuEPO DNA aptamers by successfully employing a post-SELEX strategy to identify the minimal binding core while retaining the full-length parent aptamer’s binding affinity^31^.

We have demonstrated that the panel of four anti-rHuEPO modified aptamers are specific to rHuEPO and can distinguish between Hu- and MuEPO (which have 78% amino acid sequence identity). Importantly, all modified aptamers used in this study recognize conformational epitopes; i.e. their binding affinity to thermally treated rHuEPO is compromised.

We determined the binding kinetics of the well-characterized SOMAmer reagents to Epogen/Procrit and compared these to three US and non-US-licensed rHuEPO biosimilar products, and eight non-licensed rHuEPO products. Since US-Epogen and US-Procrit are the same product manufactured by the same entity but marketed under different names, different samples of this product were tested to determine the differences in binding kinetics that naturally exists between batches of the same biological product. No significant differences (all adjusted p values > 0.10) were found between the binding kinetics of US-Epogen and US-Procrit. Overall, 3 of the 8 non-licensed rHuEPO products showed significantly different binding kinetics with 2 or more SOMAmers. These products are likely to have conformational epitopes that are different from those on Epogen/Procrit. The US and non-US-licensed rHuEPO biosimilar products tested demonstrated comparable binding kinetics to the SOMAmer reagent panel compared to Epogen/Procrit.

The finding that compared to Epogen/Procrit, some non-licensed rHuEPO products which show significant differences in binding kinetics to the SOMAmer panel is consistent with previous studies^26,27^ which showed structural and biochemical differences between the corresponding reference rHuEPO product and non-licensed rHuEPO products. The products were compared with respect to product quality attributes such as glycoforms present, the relative degree of unfolding, in vitro potency, presence of covalent aggregates, and presence of cleavage products. Of particular interest vis-a-vis our study is the conformational change monitored using the 9G8A monoclonal antibody^27^. The linear epitope recognized by the antibody contains two amino acid residues that upon proper folding are buried internal to the protein and inaccessible to the antibody; however, conformational changes or unfolding expose the epitope. Using this method the non-licensed rHuEPO products showed increased misfolding compared to the reference product^27^. Similarly, another study found high aggregate levels and protein fragments in non-licensed rHuEPO products^32^. Aggregation can be linked to misfolded protein molecules.

Taken together, there are several lines of evidence suggesting that non-licensed rHuEPO products may deviate from the conformation of the reference product. While this does not necessarily mean that the discrepancies have clinical consequences, it is important to have methods in place to detect such discrepancies. Consistent product quality and analytical similarity between reference product and biosimilar molecules are necessary to ensure that biological products are safe, pure and potent. Previous studies have discussed the advantages of aptamers over antibodies as reagents to monitor the conformational integrity/similarity of therapeutic proteins^1,2^. Briefly: (1) unlike antibodies, aptamers are not generated in an animal system and thus allow for superior quality control and can cover epitopes (regions of the protein) to which antibodies are not generated; (2) aptamers are chemically synthesized nucleic acids and thus do not have the issues related to biological reagents such as loss of potency, batch-to-batch variation and the ease with which specific functional groups can be introduced in a site-specific manner. Here we use SOMAmer reagents which have the additional advantage of side-chain modifications capable of refining interaction interfaces to suit the intended application. The incorporation of chemically diverse side chains capable of probing various surface features are readily introduced through synthetic processes. Moreover, we use these affinity reagents in conjuction with the BLI platform which is amenable to automation, allowing for high-throughput screening and measurement of kinetic parameters in solution phase. Thus, we obtain robust quantitative measurements that can be used to identify statistically significant changes associated with several epitopes on rHuEPO molecules.

Since our original study demonstrating the utility of aptamers in detecting subtle modifications in therapeutic proteins, the technique has found several applications^2,22^. Here we have revised the method to use SOMAmer reagents in lieu of conventional aptamers and the high-throughput BLI platform. This study has used reagents that target rHuEPO and we have screened different rHuEPO products, comparing them to the US-licensed reference product epoetin alfa [Epogen/Procrit]. The methodology and statistical analyses described here can be readily applied to analytically characterize other biosimilars and monitor the fidelity of the manufacturing process. In addition, drug manufacturing and distribution networks are becoming globalized and manufacturing processes are frequently transferred to different locations, often on different continents. SOMAmer-based comparisions of conformational integrity could provide an additional method in protocols that ensure that the product manufactured in a new facility is comparable to that at existing facilities. Finally, the WHO^34,35^ and others^30–32^ have reported substandard and counterfeit medical products (particulary in low- and middle-income countries) pose a serious threat that is increasing year over year. SOMAmer reagent panels offer a rapid, reliable and inexpensive tool to develop kits to identify substandard and counterfeit biologicals.

## Materials and methods

### Selection of SOMAmer reagents

Four SOMAmer reagents targeting recombinant human erythropoietin (rHuEPO) expressed in Chinese Hamster Ovary (CHO) cells (VWR, cataolg #80602-474) were discovered via the SELEX process^5,33^ from libraries containing a 40-nucleotide random region in which dT was substituted with either BndU (SL5001), TrpdU (SL5002), or NapdU (SL5003 and SL5004). The 40-nucleotide random region was flanked by a 15 or 17 nucleotide forward primer [5′ GGCAGTCCGTCCGT (SL5001) or 5′ CGCCCTCGTCCCATCTC (SL5002-SL5004)] and a 17 or 16 nucleotide reverse primer [5′ CGCCCTCGTCCCATCTC (SL5001) or 5′ GACGAGACAGGGACAG (SL5002-SL5004)]. The total length of each SELEX library was either 80 or 81 nucleotides including an eight nucleotide poly-dA region on the 3′ end. Recombinant human EPO was biotinylated for capturing protein/DNA complexes on MyOne-SA beads (Invitrogen, catalog #65001) by covalent coupling of EZ-Link NHS-PEG4-biotin (Pierce) according to the manufacturer’s instructions. Briefly, rHuEPO was mixed with fourfold molar excess of EZ-Link NHS-PEG4-biotin and incubated overnight at 4 °C in 1XSB18T buffer (40 mM HEPES, pH 7.5, 102 mM NaCl, 5 mM KCl, 5 mM MgCl_2_, 0.05% Tween-20). Excess biotin was removed using a G25 microspin column (GE Healthcare) or a Zeba column (Thermo Fisher, catalog #89883). SELEX was performed in either 1XSB17T [40 mM HEPES, pH 7.5, 102 mM NaCl, 5 mM KCl, 1 mM EDTA, 5 mM MgCl_2_, 0.05% Tween-20 (SL5001)] or 1XSB18T [40 mM HEPES, pH 7.5, 102 mM NaCl, 5 mM KCl, 5 mM MgCl_2_, 0.05% Tween-20 (SL5002-SL5004)] buffers. To preferentially select for modified aptamers with slow off-rates, a kinetic challenge was included, whereby protein/DNA complexes were incubated at 37 °C in the presence of the polyanionic competitor, dextran sulfate. The duration of the kinetic challenge was increased from 5 min in round 4 to 15 min in rounds 5–7 (SL5001) and from 5 min in rounds 5 and 6 to 30 min in rounds 7 and 8 (SL5002–SL5004). Simultaneous to the polyanionic competitor challenge, the protein concentration was lowered. SELEX was executed as follows. SELEX libraries were heat/cooled at 95 °C for 5 min, 48 °C for 5 min and 37 °C for 5 min. Following heat/cool, libraries were incubated at 37 °C for 10 min with Protein Competitor Buffer (10 μM prothrombin, 10 μM casein, 0.01% human serum albumin) and 20 μg Dynal MyOne streptavidin beads for counter selection. After 10 min the supernatant was removed and transferred to a clean plate. For round one of SELEX only, 50 pmol of protein was immobilized on 0.5 mg Dynal MyOne streptavidin beads and transferred to the counter selected library and incubated at 37 °C for 1 h with shaking. For rounds 2–7 (or 8), proteins were in solution during library incubation at 37 °C for 10 min with no shaking. Prior to capturing protein–DNA complexes with Dynal MyOne streptavidin beads for rounds 2–7 (or 8), the kinetic challenge was initiated with 5 mM dextran sulfate (final concentration). Beads were then washed two times in 25 μM biotin followed by five times in 1XSB17T or 1XSB18T buffer. Elution was achieved with 2 mM NaOH and neutralized with HCl and buffered to pH 7.5 with Tris followed by QPCR for 30 cycles, or until samples plateaued. After QPCR, DNA was captured on Dynal MyOne streptavidin beads and the sense strand eluted with 20 mM NaOH and discarded. The modified nucletodide sense strand was prepared with the appropriate nucleotide composition by primer extension from the immobilized antisense strand. After 7 (SL5001) or 8 (SL5002–SL5004) rounds of SELEX, the converged pools were sequenced.

### Modified aptamer synthesis

Modified aptamers were produced by conventional solid phase oligonucleotide synthesis using the phosphoramidite method^34^. The modified deoxyuridine-5-carboxamide phosphoramidite reagent used for solid-phase synthesis was prepared by: condensation of 5′*-O*-(4,4′-dimethoxytrityl)-5-trifluoroethoxycarbonyl-2′-deoxyuridine^41^ with the appropriate [benzylamine, 1-naphthylenemethylamine or tryptamine] primary amine (RNH_2_,1.2 eq; Et3N, 2 eq.; acetonitrile; 60 °C; 4 h); 3′-*O*-phosphitylation with 2-cyanoethyl-*N*,*N*,*N*′,*N*′-tetraisopropylphosphene (1.05 eq; pyridine trifluoroacetate, 1.1 eq; CH_2_Cl_2_; room temperature, approximately 1 h); and purification by flash chromatography on neutral silica gel^42^. Modified aptamers were synthesized at the 1 µmole scale on an ABI 3900 DNA synthesizer with some adjustments to the protocol to account for the unique base modification described herein. Detritylation was accomplished with 10% dichloroacetic acid in toluene; coupling was achieved with 0.1 M phosphoramidites in straight acetonitrile or a mix of acetonitrile:dichloromethane activated by 5-benzylmercaptotetrazole and allowed to react 3 times; capping and oxidation were performed according to instrument vendor recommendations. Deprotection was effected with aqueous *t*-butylamine/methanol^43^ using an optimized time and temperature. Products were collected by filtration and evaporated in a Genevac HT-12, redissolved in di water and purified by preparative ion-pairing reversed phase liquid chromatography, evaporated to dryness in a Genevac HT-12 system, redissolved in di water and desalted on GE Hi-Trap Sephadex G-25 columns. The purified product was characterized by Ultra Performance Liquid Chromatography (UPLC, Waters Acquity system), mass spectrometry (Agilent 1100 with Bruker Ion-trap detector), capillary gel electrophoresis (Beckman Coulter P/ACE CGE), UV spectrophotometry, and protein binding affinity in buffered aqueous solution.

### Characterization of modified aptamers

Equilibrium affinity rate constants (K_D_ values) of synthetic 50-mer modified aptamers (40 nucleotides from the SELEX library modified region and five nucleotides from the 5′ and 3′ primer regions) were determined by filter binding assay (5′-OH reagents). K_D_ values of modified aptamers were measured in SB18T buffer. Modified aptamers were 5′ end labeled using T4 polynucleotide kinase (New England Biolabs) and γ-[^32^P]ATP (Perkin-Elmer). Radiolabeled aptamers (~ 20,000 CPM (filter binding) were mixed with rHuEPO at concentrations ranging from 10^–7^ to 10^–12^ M and incubated at 37 °C for 40 min. Bound complexes were partitioned on MyOne streptavidin beads and captured on Durapore filter plates (EMD Millipore). The fraction of bound aptamer was quantified with a phosphorimager (Fujifilm FLA-3000) and data were analyzed in Image Gauge version 4.0 (Fujifilm). To determine binding affinity, data were fit using the equation:

$$ {\text{y } = \text{ }}\left( {{\max} - {\min}} \right)\left( {{\text{Protein}}} \right)/\left( {K_{D} + {\text{Protein}}} \right) + {\min} $$

and plotted using GraphPad Prism version 7.00.

### Biochemical characterization of the erythropoietin molecules

For Western blot analysis, 30 IU of rHuEPO products were diluted in reducing sample buffer, separated on 4–20% gradient gels, transferred to nitrocellulose membranes using Trans-Blot Turbo system (Bio-Rad) and blocked with 5% skim milk in TPBS buffer (PBS buffer with Tween 20). Membrane-bound proteins were probed with rabbit polyclonal IgG specific to human EPO (EPO (H-162), sc-7956, 1:1000) overnight, followed by incubation with appropriate secondary antibodies (Abcam Goat Anti-Rabbit IgG H&L, 1:10,000). The membrane was then imaged and analyzed using an Image Station 4000MM PRO (Carestream) with Carestream MI software version 4.5 (https://www.bruker.com/service/support-upgrades/software-downloads/molecular-imaging.html). Images were captured within the linear range for each probe.

For thermal stability studies, rHuEPO [Epogen/Procrit] was diluted to 10 µg/mL in PBS and incubated in a 60 °C water bath for 0, 1, or 17 h. Following heat treatment, rHuEPO samples were frozen at − 80 °C until the time of assay.

### High throughput bio-layer interferometry (BLI)

Biomolecular binding kinetics parameters of interactions between rHuEPO products and modified aptamers were acquired using an Octet RED96 instrument and Octet System Data Acquisition software version 8.2 (Pall ForteBio; https://www.fortebio.com/products/octet-systems-software). Prior to BLI analysis, modified aptamers were refolded upon heat treatment at 98 °C for 3 min followed by incubation at room temperature for at least 10 min. Biotinylated aptamers (50 nM) were then immobilized on steptavidin-coated biosensors (ForteBio #18-5019). rHuEPO products were diluted to desired concentrations in binding buffer (PBS + 0.02% Tween 20) and tested for binding (association and dissociation) to immobilized aptamers.

### Statistical analyses

Biomolecular binding kinetics parameters (association rate constant (k_a_) [1/Ms]; dissociation rate constant (k_d_) [1/s]; affinity rate constant (K_D_) [M]) were analyzed using Octet System Data Analysis software version 8.2 (Pall ForteBio; https://www.fortebio.com/products/octet-systems-software). Only data sets with curve fit R^2^ values greater than 0.95 were included in further analyses as per the manufacturer’s recommendation.

Significance of differences between products and Epogen/Procrit with respect to k_a_, k_d_, or K_D_ was conducted using two-sided t test and adjusted for multiple comparisons using the Benjamini, Hochberg method with a false positive cutoff value of 0.10.

Testing the significance of fold change of K_D_ comparing rMuEPO with rHuEPO was achieved through One-Way ANOVA and controlled for variations due to SOMAmer using multplie regressions.

### Disclaimer

Our contributions are an informal communication and represent our own best judgement. These comments do not bind or obligate FDA.

## Supplementary information

Supplementary Information.

Supplementary Table 2.

Supplementary Table 3.
