# Supplementary material for: Modified aptamers as reagents to characterize recombinant human erythropoietin products
Source: Sci Rep. 2020 Oct 29;10:18593. doi: 10.1038/s41598-020-75713-2 (PMC7596557; doi:10.1038/s41598-020-75713-2)
Supplement: Supplementary file 1 — Supplementary Information. [file 41598_2020_75713_MOESM1_ESM.docx]

**Supplementary Information**

**Modified aptamers as reagents to characterize recombinant human erythropoietin products**

Wojciech Jankowski^1*^, H.A. Daniel Lagassé^1*^, William C. Chang^1^, Joseph McGill^1^, Katarzyna I. Jankowska^2^, Amy D. Gelinas^3^, Nebojsa Janjic^3*^ and Zuben E. Sauna^1*^

^1^Hemostasis Branch, Division of Plasma Protein Therapeutics, Center for Biologics Evaluation and Research, Food and Drug Administration, Silver Spring, MD, USA. ^2^Laboratory of Cellular Hematology, Division of Blood Components and Devices, Center for Biologics Evaluation and Research, Food and Drug Administration, Silver Spring, MD, USA. ^3^SomaLogic, Inc., Boulder, CO USA.

* Authors contributed equally to this work and are listed in alphabetical order.

Correspondence: [zuben.sauna@fda.hhs.gov](mailto:zuben.sauna@fda.hhs.gov) (Z.E. Sauna) or [njanjic@somalogic.com](mailto:njanjic@somalogic.com) (N. Janjic)

**
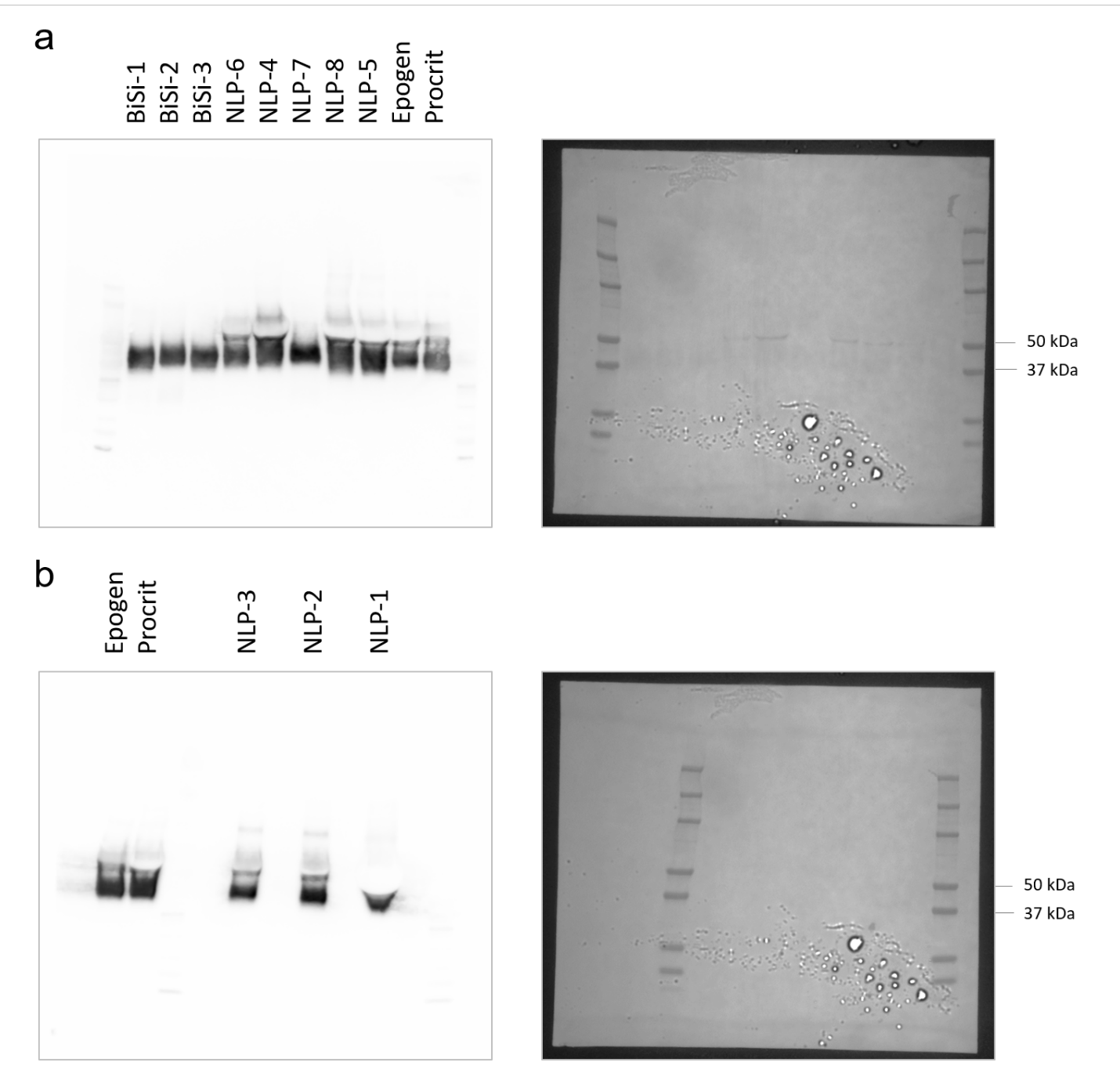
Supplementary Figure 1. Full-length blots quantified in Figure 2.**

Western Blot of rHuEPO products: (a) BiSi 1-3, NLP 6-8, and (b) NLP 1-3 referred to Epogen and Procrit. The molecular weight of the EPO bands at ~37 kDa were determined by Precision Plus Protein Dual Color Standards (Bio-Rad).

**Supplementary Table 1. Binding affinities.** Comparison of median binding affinities (K_D_ values) of the four SOMAmers binding rHuEPO and rMuEPO by filter binding and BLI methods.

|  | **Median K_D_ (M)** | | |
| --- | --- | --- | --- |
|  | **rHuEPO** | | **rMuEPO** |
| **SOMAmer** | **filter binding** | **BLI** | **BLI** |
| SL5001 | 3.5 x 10^-9^ | 2.1 x 10^-9^ | 5.6 x 10^-10^ |
| SL5002 | 8.2 x 10^-9^ | 5.2 x 10^-9^ | 4.4 x 10^-10^ |
| SL5003 | 2.8 x 10^-10^ | 4.4 x 10^-10^ | 1.3 x 10^-10^ |
| SL5004 | 2.3 x 10^-10^ | 9.4 x 10^-10^ | 1.8 x 10^-10^ |

**Supplementary Table 2** and **Supplementary Table 3** are provided as separate documents.

**Supplementary Table 2.** Summary of all kinetic measurements presented in this study. Biomolecular binding kinetics parameters (association rate constant (k_a_) [1/Ms]; dissociation rate constant (k_d_) [1/s]; affinity rate constant (K_D_) [M]) were analyzed using Octet System Data Analysis software version 8.2 (Pall ForteBio; <https://www.fortebio.com/products/octet-systems-software>).

**Supplementary Table 3.** Comparison of binding kinetics of each product with the respective Epogen/Procrit sample. Significance of differences between products and Epogen/Procrit with respect to ka, kd, or KD was conducted using two-sided t-test and adjusted for multiple comparisons using the Benjamini, Hochberg method with a false positive cutoff value of 0.10.
