# Supplementary material for: Modified aptamers as reagents to characterize recombinant human erythropoietin products
Source: Sci Rep. 2020 Oct 29;10:18593. doi: 10.1038/s41598-020-75713-2 (PMC7596557; doi:10.1038/s41598-020-75713-2)
Supplement: Supplementary file 2 — Supplementary Table 2. [file 41598_2020_75713_MOESM2_ESM.pdf]

Supplemental Table 2:

SOMAmer	Product	KD		ka		kd	
		KD	Error	ka	Error	kd	Error
SL5001	BiSi-1	2.27e-09	1.48e-11	1.14e+05	615	9.5e-07	1.00e-06
SL5001	BiSi-1	1.25e-09	1.53e-11	2.76e+05	2900	2.16e-06	2.20e-06
SL5001	BiSi-1	4.30e-09	1.17e-10	8.18e+04	2740	1.45e-06	1.50e-06
SL5001	BiSi-1	1.07e-09	3.12e-11	2.40e+05	8440	2.05e-06	2.00e-06
SL5001	BiSi-1	1.72e-09	5.76e-10	4.84e+04	20000	2.26e-06	2.30e-06
SL5001	BiSi-2	2.15e-09	1.72e-11	1.18e+05	767	1.17e-06	1.20e-06
SL5001	BiSi-2	2.00e-09	2.97e-11	2.11e+05	2860	2.52e-06	2.50e-06
SL5001	BiSi-3	2.57e-09	2.06e-11	1.17e+05	797	1.25e-06	1.30e-06
SL5001	BiSi-3	1.83e-09	1.77e-11	1.94e+05	1690	1.47e-06	1.50e-06
SL5001	BiSi-3	2.81e-09	4.34e-11	1.45e+05	2710	1.43e-06	1.40e-06
SL5001	BiSi-3	2.51e-09	5.74e-10	2.42e+04	6770	1.51e-06	1.50e-06
SL5001	Epogen	2.43e-09	1.82e-11	102900	642.8	1.03e-06	1.00e-06
SL5001	Epogen	1.35e-09	1.49e-11	219400	2075	1.67e-06	1.70e-06
SL5001	Epogen	4.89e-10	7.64e-12	580400	7384	2.58e-06	2.60e-06
SL5001	Epogen	5.34e-09	5.24e-11	29100	171	1.22e-06	1.20e-06
SL5001	Epogen	3.23e-09	2.54e-11	47200	218	9.73e-07	1.00e-06
SL5001	Epogen	2.91e-09	1.49e-11	57400	194	6.38e-07	6.00e-07
SL5001	Epogen	2.57e-09	1.43e-11	66000	288	5.85e-07	6.00e-07
SL5001	Epogen	2.03e-09	3.66e-11	67600	1110	1e-06	1.00e-06
SL5001	Epogen	2.27e-09	1.68e-11	1.08e+05	656	1.03e-06	1.00e-06
SL5001	Epogen	1.19e-09	1.27e-11	2.38e+05	2140	1.65e-06	1.60e-06
SL5001	Epogen	1.98e-09	1.23e-10	5.35e+04	3970	1.8e-06	1.80e-06
SL5001	Epogen	1.42e-08	9.82e-09	1.22e+04	10400	2.5e-06	2.50e-06
SL5001	Mouse	5.64e-10	1.77e-12	2.54e+05	639	2.7e-07	3.00e-07
SL5001	Mouse	6.08e-10	3.59e-12	2.59e+05	1430	3.27e-07	3.00e-07
SL5001	Mouse	4.61e-10	5.53e-12	3.61e+05	4190	4.9e-07	5.00e-07
SL5001	NLP-1	8.95e-10	9.91e-12	171000	1110	1.37e-06	1.40e-06
SL5001	NLP-1	5.64e-10	3.75e-12	154000	598	4.7e-07	5.00e-07
SL5001	NLP-1	5.99e-10	1.3e-11	125000	2200	9.53e-07	1.00e-06
SL5001	NLP-2	6.57e-10	7.02e-12	3.46e+05	3130	1.3e-06	1.30e-06
SL5001	NLP-2	1.07e-09	2.90e-11	2.20e+05	5800	1.41e-06	1.40e-06
SL5001	NLP-2	1.10e-08	8.73e-09	2.19e+04	17400	2.2e-06	2.20e-06
SL5001	NLP-3	5.35e-09	2.55e-11	33900	108	6.46e-07	6.00e-07
SL5001	NLP-3	4.69e-09	2.09e-11	40400	139	5.39e-07	5.00e-07
SL5001	NLP-3	5.91e-09	6.89e-11	30200	328	7.51e-07	8.00e-07
SL5001	NLP-4	1.57e-09	9.32e-12	1.32e+05	582	8.19e-07	8.00e-07
SL5001	NLP-4	9.09e-10	9.71e-12	2.13e+05	1740	1.33e-06	1.30e-06
SL5001	NLP-4	1.70e-09	2.61e-11	1.37e+05	2490	1.18e-06	1.20e-06
SL5001	NLP-4	1.44e-08	8.47e-09	1.21e+04	8850	2.14e-06	2.10e-06
SL5001	NLP-5	1.08e-09	1.03e-11	1.67e+05	1060	1.28e-06	1.30e-06
SL5001	NLP-5	1.87e-09	2.11e-11	1.51e+05	2020	9.81e-07	1.00e-06
SL5001	NLP-5	6.46e-10	1.11e-11	3.00e+05	5940	1.36e-06	1.40e-06
SL5001	NLP-5	4.91e-10	1.79e-11	3.17e+05	13800	1.6e-06	1.60e-06
SL5001	NLP-6	2.21e-09	2.35e-11	1.46e+05	1310	1.86e-06	1.90e-06
SL5001	NLP-6	1.22e-09	1.50e-11	3.01e+05	3200	2.3e-06	2.30e-06
SL5001	NLP-6	1.15e-09	1.28e-11	2.36e+05	2960	1.35e-06	1.40e-06
SL5001	NLP-6	8.69e-10	3.47e-11	2.14e+05	10100	2.36e-06	2.40e-06
SL5001	NLP-7	1.77e-09	8.89e-12	1.13e+05	431	6.47e-07	6.00e-07
SL5001	NLP-7	9.87e-10	8.48e-12	2.01e+05	1350	1.06e-06	1.10e-06
SL5001	NLP-7	4.76e-10	6.19e-12	5.31e+05	5510	1.98e-06	2.00e-06
SL5001	NLP-7	2.85e-09	4.99e-11	6.98e+04	1480	7.13e-07	7.00e-07
SL5001	NLP-7	1.19e-08	2.87e-09	1.72e+04	5110	1.26e-06	1.30e-06
SL5001	NLP-8	1.80e-09	7.51e-12	1.21e+05	390	5.75e-07	6.00e-07
SL5001	NLP-8	9.40e-10	6.93e-12	2.23e+05	1280	9.7e-07	1.00e-06
SL5001	NLP-8	3.78e-10	4.68e-12	5.10e+05	4610	1.63e-06	1.60e-06
SL5001	NLP-8	1.06e-08	6.44e-10	2.29e+04	1710	8.63e-07	9.00e-07
SL5001	NLP-8	2.44e-08	1.67e-08	9.32e+03	7930	1.99e-06	2.00e-06
SL5001	Procrit	4.07e-09	4.24e-11	35200	215	1.21e-06	1.20e-06
SL5001	Procrit	3.03e-09	2.1e-11	49100	197	8.41e-07	8.00e-07
SL5001	Procrit	2.67e-09	1.23e-11	63900	193	5.95e-07	6.00e-07
SL5001	Procrit	2.33e-09	1.23e-11	73600	297	5.87e-07	6.00e-07
SL5001	Procrit	2.46e-09	4.11e-11	88500	1380	1.28e-06	1.30e-06
SL5001	Procrit	2.13e-09	1.48e-11	1.08e+05	605	9.39e-07	9.00e-07
SL5001	Procrit	1.38e-09	1.15e-11	1.86e+05	1330	1.1e-06	1.10e-06
SL5001	Procrit	3.26e-08	2.20e-08	5.58e+03	3760	1.83e-06	1.80e-06
SL5002	BiSi-1	2.54e-09	3.09e-11	2.08e+05	2270	2.86e-06	2.90e-06
SL5002	BiSi-1	7.01e-10	1.33e-11	1.64e+05	3180	1.38e-06	1.40e-06
SL5002	BiSi-1	1.13e-08	1.14e-08	7.89e+03	9860	2.25e-06	2.20e-06
SL5002	BiSi-2	3.57e-09	2.78e-11	1.29e+05	910	1.51e-06	1.50e-06
SL5002	BiSi-2	1.77e-09	1.63e-11	2.68e+05	2240	1.83e-06	1.80e-06
SL5002	BiSi-2	3.53e-10	6.54e-12	2.94e+05	4240	1.66e-06	1.70e-06
SL5002	BiSi-2	3.05e-10	8.08e-12	3.81e+05	9830	2.09e-06	2.10e-06
SL5002	BiSi-3	9.18e-10	3.18e-11	1.24e+05	4680	2.07e-06	2.10e-06
SL5002	BiSi-3	#VALUe!	1.64e-10	#VALUe!	12000	NA	NA
SL5002	Epogen	5.54e-09	8.14e-11	121100	1680	3.26e-06	3.30e-06
SL5002	Epogen	4.92e-09	1.03e-10	232800	4687	6.16e-06	6.20e-06
SL5002	Epogen	1.63e-09	4.51e-11	924000	24280	1.29e-05	1.29e-05
SL5002	Epogen	2.15e-08	1.36e-10	14600	74.8	1.18e-06	1.20e-06
SL5002	Epogen	1.71e-08	1.05e-10	19400	97.8	1.16e-06	1.20e-06
SL5002	Epogen	1.2e-08	7e-11	27800	139	1.02e-06	1.00e-06
SL5002	Epogen	8.82e-09	4.78e-11	37100	180	7.81e-07	8.00e-07
SL5002	Epogen	5.01e-09	3.74e-11	60800	414	9.33e-07	9.00e-07
SL5002	Epogen	2.70e-09	3.22e-11	1.86e+05	1980	2.69e-06	2.70e-06
SL5002	Mouse	5.91e-10	4.86e-12	4.22e+05	2890	1.14e-06	1.10e-06
SL5002	Mouse	4.38e-10	4.08e-12	7.02e+05	5860	1.28e-06	1.30e-06
SL5002	Mouse	1.60e-10	1.87e-12	1.29e+06	12600	1.32e-06	1.30e-06
SL5002	NLP-1	1.6e-09	1.34e-11	89400	494	8.99e-07	9.00e-07
SL5002	NLP-1	1.4e-09	1.28e-11	120000	840	1e-06	1.00e-06
SL5002	NLP-1	1.47e-09	1.54e-11	137000	1250	1.07e-06	1.10e-06
SL5002	NLP-2	9.61e-10	1.20e-11	3.50e+05	3970	1.76e-06	1.80e-06
SL5002	NLP-2	6.71e-10	1.30e-11	5.50e+05	10100	2.41e-06	2.40e-06
SL5002	NLP-3	1.3e-08	9.57e-11	20800	131	1.05e-06	1.00e-06
SL5002	NLP-3	1.23e-08	1.57e-10	21400	255	1.19e-06	1.20e-06
SL5002	NLP-3	2.2e-08	9.89e-10	11100	495	1.24e-06	1.20e-06
SL5002	NLP-4	1.02e-08	2.89e-10	2.80e+05	7680	1.92e-05	1.92e-05
SL5002	NLP-5	3.48e-09	3.67e-11	1.49e+05	1430	2.26e-06	2.30e-06
SL5002	NLP-5	8.88e-09	1.19e-09	1.88e+04	3100	1.49e-06	1.50e-06
SL5002	NLP-5	1.17e-08	5.28e-09	1.43e+04	7990	1.92e-06	1.90e-06
SL5002	NLP-5	2.72e-09	1.75e-09	2.92e+04	23200	2.63e-06	2.60e-06
SL5002	NLP-6	3.20e-08	1.54e-09	7.65e+04	3640	1.95e-05	1.95e-05
SL5002	NLP-6	9.83e-09	4.07e-10	3.11e+05	12500	3.12e-05	3.12e-05
SL5002	NLP-6	1.21e-09	3.00e-11	1.50e+05	4250	1.93e-06	1.90e-06
SL5002	NLP-7	4.07e-09	3.46e-11	1.33e+05	1050	1.79e-06	1.80e-06
SL5002	NLP-7	2.64e-09	3.57e-11	3.79e+05	4830	4.43e-06	4.40e-06
SL5002	NLP-7	1.19e-09	2.11e-11	2.11e+05	4250	1.94e-06	1.90e-06
SL5002	NLP-8	6.24e-09	8.18e-11	1.39e+05	1730	3.54e-06	3.50e-06
SL5002	NLP-8	2.85e-09	3.76e-11	2.89e+05	3600	3.52e-06	3.50e-06
SL5002	NLP-8	6.62e-10	9.45e-12	2.46e+05	3540	1.49e-06	1.50e-06
SL5002	NLP-8	3.02e-10	5.11e-12	5.53e+05	9210	1.9e-06	1.90e-06
SL5002	Procrit	3.58e-08	2.09e-10	10800	53.8	1.2e-06	1.20e-06
SL5002	Procrit	2.28e-08	1.27e-10	16400	77.9	1.07e-06	1.10e-06
SL5002	Procrit	1.89e-08	9.35e-11	21600	96.9	8.38e-07	8.00e-07
SL5002	Procrit	1.89e-08	1.6e-10	24800	202	1.05e-06	1.00e-06
SL5002	Procrit	1.09e-07	1.35e-08	5190	644	2.03e-06	2.00e-06
SL5002	Procrit	8.54e-09	1.58e-10	1.79e+05	3170	8.21e-06	8.20e-06
SL5002	Procrit	2.37e-09	4.18e-11	4.88e+05	8140	6.7e-06	6.70e-06
SL5002	Procrit	7.79e-09	3.18e-10	5.12e+05	20400	3.36e-05	3.36e-05
SL5003	BiSi-1	5.17e-10	4.89e-12	6.78e+05	5370	1.81e-06	1.80e-06
SL5003	BiSi-1	4.42e-10	3.90e-12	6.61e+05	4700	1.53e-06	1.50e-06
SL5003	BiSi-1	7.98e-10	5.67e-12	4.71e+05	3630	1.42e-06	1.40e-06
SL5003	BiSi-1	1.11e-09	8.54e-12	2.11e+05	1950	4.71e-07	5.00e-07
SL5003	BiSi-1	6.00e-09	1.08e-09	2.89e+04	6400	7.74e-07	8.00e-07
SL5003	BiSi-2	4.74e-10	3.37e-12	7.93e+05	5870	1.6e-06	1.60e-06
SL5003	BiSi-2	5.12e-10	3.71e-12	6.09e+05	4920	1.08e-06	1.10e-06
SL5003	BiSi-2	1.14e-09	2.36e-11	1.90e+05	4820	5.91e-07	6.00e-07
SL5003	BiSi-3	6.48e-10	7.20e-12	5.49e+05	5340	1.9e-06	1.90e-06
SL5003	BiSi-3	5.69e-10	5.06e-12	5.77e+05	4190	1.68e-06	1.70e-06
SL5003	BiSi-3	5.45e-10	4.81e-12	5.20e+05	3800	1.41e-06	1.40e-06
SL5003	BiSi-3	1.01e-09	7.05e-12	3.23e+05	2500	1.1e-06	1.10e-06
SL5003	BiSi-3	1.05e-09	1.04e-11	1.75e+05	2070	4.88e-07	5.00e-07
SL5003	BiSi-3	2.52e-09	1.42e-10	7.42e+04	5140	6.25e-07	6.00e-07
SL5003	Epogen	5.88e-10	1.31e-11	969400	20560	3.94e-06	3.90e-06
SL5003	Epogen	4.95e-10	7.83e-12	1059000	15370	3.29e-06	3.30e-06
SL5003	Epogen	3.71e-10	4.93e-12	1184000	13700	2.88e-06	2.90e-06
SL5003	Epogen	7.81e-10	7.78e-12	407000	3690	1.32e-06	1.30e-06
SL5003	Epogen	6.42e-10	4.62e-12	443000	2670	1.11e-06	1.10e-06
SL5003	Epogen	5.93e-10	3.3e-12	405000	1670	9e-07	9.00e-07
SL5003	Epogen	1.13e-09	8.76e-12	160000	1150	5.26e-07	5.00e-07
SL5003	Epogen	1.09e-09	4.67e-11	108000	4510	1.03e-06	1.00e-06
SL5003	Epogen	4.42e-10	3.26e-12	8.57e+05	6580	1.67e-06	1.70e-06
SL5003	Epogen	5.33e-10	2.53e-12	7.03e+05	3740	8.2e-07	8.00e-07
SL5003	Epogen	3.28e-10	4.79e-12	7.23e+05	12100	1.4e-06	1.40e-06
SL5003	Mouse	9.51e-11	1.03e-12	1.72e+06	12400	1.34e-06	1.30e-06
SL5003	Mouse	1.67e-10	2.00e-12	1.24e+06	12600	1.33e-06	1.30e-06
SL5003	NLP-1	6.35e-10	4.37e-12	325000	1620	9.79e-07	1.00e-06
SL5003	NLP-1	6.13e-10	4.18e-12	312000	1420	9.77e-07	1.00e-06
SL5003	NLP-1	6.22e-10	3.48e-12	284000	1090	7.23e-07	7.00e-07
SL5003	NLP-2	4.27e-11	<1.0e-12	9.82e+06	141000	2.1e-06	2.10e-06
SL5003	NLP-2	3.65e-11	<1.0e-12	1.08e+07	92000	1.59e-06	1.60e-06
SL5003	NLP-3	1.7e-09	1.26e-11	168000	1100	1.01e-06	1.00e-06
SL5003	NLP-3	1.42e-09	9.2e-12	188000	985	1.02e-06	1.00e-06
SL5003	NLP-3	1.39e-09	8.15e-12	192000	859	1.01e-06	1.00e-06
SL5003	NLP-4	4.09e-10	3.27e-12	9.48e+05	7920	1.83e-06	1.80e-06
SL5003	NLP-4	3.77e-10	2.36e-12	7.42e+05	4920	1e-06	1.00e-06
SL5003	NLP-4	9.26e-10	1.18e-11	2.97e+05	4620	5.83e-07	6.00e-07
SL5003	NLP-5	3.36e-10	3.14e-12	1.23e+06	12400	2.11e-06	2.10e-06
SL5003	NLP-5	3.61e-10	3.68e-12	8.38e+05	9920	1.17e-06	1.20e-06
SL5003	NLP-6	6.60e-10	1.41e-11	2.21e+06	45000	9.38e-06	9.40e-06
SL5003	NLP-6	5.78e-10	5.69e-12	8.92e+05	9640	2.57e-06	2.60e-06
SL5003	NLP-6	6.15e-10	4.78e-12	6.16e+05	5460	1.25e-06	1.30e-06
SL5003	NLP-6	1.18e-09	2.22e-11	2.91e+05	6660	8.81e-07	9.00e-07
SL5003	NLP-7	4.59e-10	4.35e-12	7.35e+05	5830	1.76e-06	1.80e-06
SL5003	NLP-7	4.23e-10	3.92e-12	7.41e+05	5590	1.69e-06	1.70e-06
SL5003	NLP-7	5.81e-10	3.89e-12	6.06e+05	4230	1.4e-06	1.40e-06
SL5003	NLP-7	8.49e-10	4.52e-12	3.27e+05	2050	4.92e-07	5.00e-07
SL5003	NLP-7	4.61e-10	6.30e-12	3.81e+05	6180	7.21e-07	7.00e-07
SL5003	NLP-8	4.40e-10	4.75e-12	7.77e+05	7080	1.99e-06	2.00e-06
SL5003	NLP-8	4.17e-10	3.91e-12	7.23e+05	5460	1.67e-06	1.70e-06
SL5003	NLP-8	5.75e-10	3.62e-12	5.48e+05	3540	1.22e-06	1.20e-06
SL5003	NLP-8	7.64e-10	4.62e-12	3.32e+05	2350	5.54e-07	6.00e-07
SL5003	NLP-8	3.83e-10	9.30e-12	5.29e+05	15000	1.75e-06	1.80e-06
SL5003	Procrit	9.94e-10	1.54e-11	352000	5170	1.7e-06	1.70e-06
SL5003	Procrit	8.57e-10	9.88e-12	403000	4270	1.58e-06	1.60e-06
SL5003	Procrit	8.18e-10	5.72e-12	368000	2140	1.17e-06	1.20e-06
SL5003	Procrit	6.37e-10	3.17e-12	324000	1150	7.18e-07	7.00e-07
SL5003	Procrit	9.15e-10	8.27e-12	166000	1350	6.02e-07	6.00e-07
SL5003	Procrit	4.67e-10	3.21e-12	8.11e+05	4720	1.38e-06	1.40e-06
SL5003	Procrit	4.37e-10	2.55e-12	6.56e+05	3420	7.48e-07	7.00e-07
SL5003	Procrit	2.07e-10	2.05e-12	1.02e+06	8920	9.8e-07	1.00e-06
SL5004	BiSi-1	1.14e-09	8.84e-12	3.09e+05	2000	1.51e-06	1.50e-06
SL5004	BiSi-1	7.42e-10	4.33e-12	4.37e+05	2070	1.1e-06	1.10e-06
SL5004	BiSi-1	5.82e-10	3.13e-12	5.83e+05	2670	9.61e-07	1.00e-06
SL5004	BiSi-1	1.12e-09	3.17e-12	3.54e+05	1130	5.02e-07	5.00e-07
SL5004	BiSi-1	9.71e-10	4.36e-12	3.99e+05	2120	5.34e-07	5.00e-07
SL5004	BiSi-1	1.76e-09	2.64e-11	2.46e+05	4540	6.41e-07	6.00e-07
SL5004	BiSi-2	1.17e-09	1.20e-11	3.53e+05	3140	2.1e-06	2.10e-06
SL5004	BiSi-2	7.70e-10	6.36e-12	5.27e+05	3720	1.73e-06	1.70e-06
SL5004	BiSi-2	5.49e-10	4.24e-12	7.14e+05	4740	1.55e-06	1.60e-06
SL5004	BiSi-2	9.24e-10	4.03e-12	4.69e+05	2270	9.19e-07	9.00e-07
SL5004	BiSi-2	7.33e-10	4.42e-12	5.83e+05	4080	9.77e-07	1.00e-06
SL5004	BiSi-2	9.93e-10	8.56e-12	4.37e+05	4590	6.32e-07	6.00e-07
SL5004	BiSi-3	1.09e-09	7.83e-12	2.82e+05	1620	1.32e-06	1.30e-06
SL5004	BiSi-3	7.94e-10	4.09e-12	3.95e+05	1650	9.49e-07	9.00e-07
SL5004	BiSi-3	6.61e-10	3.69e-12	5.12e+05	2470	9.52e-07	1.00e-06
SL5004	BiSi-3	1.23e-09	4.04e-12	3.20e+05	1200	5.48e-07	5.00e-07
SL5004	BiSi-3	1.37e-09	8.92e-12	2.84e+05	2230	5.85e-07	6.00e-07
SL5004	BiSi-3	1.05e-09	1.10e-11	3.91e+05	5010	6.83e-07	7.00e-07
SL5004	Epogen	1.33e-09	1.93e-11	420600	5568	3.32e-06	3.30e-06
SL5004	Epogen	7.48e-10	1.01e-11	681400	8186	3.06e-06	3.10e-06
SL5004	Epogen	5.04e-10	6.78e-12	1068000	12850	3.23e-06	3.20e-06
SL5004	Epogen	1.74e-09	1.68e-11	108000	691	1.36e-06	1.40e-06
SL5004	Epogen	1.53e-09	8.84e-12	183000	823	1.02e-06	1.00e-06
SL5004	Epogen	9.46e-10	4.17e-12	281000	994	6.99e-07	7.00e-07
SL5004	Epogen	1.1e-09	4.77e-12	276000	1100	5.07e-07	5.00e-07
SL5004	Epogen	1.11e-09	1.39e-11	314000	3850	9.83e-07	1.00e-06
SL5004	Epogen	9.22e-10	3.73e-12	3.89e+05	1730	7.27e-07	7.00e-07
SL5004	Epogen	9.33e-10	5.93e-12	3.79e+05	2850	7.07e-07	7.00e-07
SL5004	Epogen	6.51e-10	1.02e-11	4.81e+05	9130	1.15e-06	1.20e-06
SL5004	Mouse	2.73e-10	4.44e-12	4.58e+05	4530	1.62e-06	1.60e-06
SL5004	Mouse	8.93e-11	2.48e-12	7.74e+05	9400	1.72e-06	1.70e-06
SL5004	NLP-1	1.01e-09	4.51e-12	225000	802	6.12e-07	6.00e-07
SL5004	NLP-1	1.02e-09	8.06e-12	229000	1650	7.49e-07	7.00e-07
SL5004	NLP-1	9.99e-10	1.36e-11	244000	3210	7.79e-07	8.00e-07
SL5004	NLP-2	2.74e-10	2.81e-12	1.94e+06	18200	2.21e-06	2.20e-06
SL5004	NLP-2	1.39e-10	1.11e-12	2.69e+06	18300	1.58e-06	1.60e-06
SL5004	NLP-2	1.05e-10	<1.0e-12	4.03e+06	20600	1.27e-06	1.30e-06
SL5004	NLP-3	2.28e-09	9.27e-12	132000	437	7.12e-07	7.00e-07
SL5004	NLP-3	1.55e-09	7.12e-12	154000	571	6.51e-07	7.00e-07
SL5004	NLP-3	2.99e-09	2.56e-11	110000	897	8.7e-07	9.00e-07
SL5004	NLP-4	1.01e-09	1.00e-11	3.77e+05	3200	1.93e-06	1.90e-06
SL5004	NLP-4	6.74e-10	4.83e-12	5.32e+05	3180	1.42e-06	1.40e-06
SL5004	NLP-4	5.03e-10	3.63e-12	7.64e+05	4690	1.45e-06	1.50e-06
SL5004	NLP-4	7.27e-10	2.98e-12	6.14e+05	2750	9.57e-07	1.00e-06
SL5004	NLP-4	6.86e-10	3.00e-12	5.60e+05	2830	6.65e-07	7.00e-07
SL5004	NLP-4	8.63e-10	7.48e-12	5.08e+05	5350	7.31e-07	7.00e-07
SL5004	NLP-5	6.48e-10	7.42e-12	7.16e+05	7260	2.47e-06	2.50e-06
SL5004	NLP-5	4.25e-10	4.37e-12	1.05e+06	9420	2.26e-06	2.30e-06
SL5004	NLP-5	5.97e-10	3.34e-12	7.10e+05	4250	1.31e-06	1.30e-06
SL5004	NLP-5	4.99e-10	2.39e-12	7.91e+05	4230	9.01e-07	9.00e-07
SL5004	NLP-5	4.54e-10	2.92e-12	9.16e+05	6950	8.7e-07	9.00e-07
SL5004	NLP-6	1.32e-09	1.58e-11	3.89e+05	4200	2.67e-06	2.70e-06
SL5004	NLP-6	8.68e-10	7.50e-12	5.27e+05	3980	1.92e-06	1.90e-06
SL5004	NLP-6	6.94e-10	5.73e-12	7.12e+05	5260	1.83e-06	1.80e-06
SL5004	NLP-6	1.10e-09	5.90e-12	4.75e+05	2890	1.23e-06	1.20e-06
SL5004	NLP-6	9.37e-10	4.64e-12	4.89e+05	2860	7.27e-07	7.00e-07
SL5004	NLP-6	3.22e-09	1.04e-10	1.79e+05	7140	1.12e-06	1.10e-06
SL5004	NLP-7	7.26e-10	8.43e-12	6.88e+05	7140	2.62e-06	2.60e-06
SL5004	NLP-7	4.83e-10	4.71e-12	9.88e+05	8490	2.21e-06	2.20e-06
SL5004	NLP-7	8.22e-10	2.61e-12	4.35e+05	1500	6e-07	6.00e-07
SL5004	NLP-7	1.18e-09	5.20e-12	3.26e+05	1720	4.43e-07	4.00e-07
SL5004	NLP-7	6.97e-10	6.60e-12	5.35e+05	6120	7.98e-07	8.00e-07
SL5004	NLP-8	6.87e-10	7.51e-12	6.65e+05	6400	2.37e-06	2.40e-06
SL5004	NLP-8	4.59e-10	4.95e-12	9.83e+05	9240	2.38e-06	2.40e-06
SL5004	NLP-8	8.59e-10	2.72e-12	4.05e+05	1400	5.73e-07	6.00e-07
SL5004	NLP-8	9.57e-10	5.67e-12	3.19e+05	2250	5.51e-07	6.00e-07
SL5004	NLP-8	6.44e-10	6.16e-12	5.27e+05	6070	7.74e-07	8.00e-07
SL5004	Procrit	2.34e-09	1.88e-11	112000	685	1.37e-06	1.40e-06
SL5004	Procrit	1.67e-09	8.72e-12	169000	687	9.19e-07	9.00e-07
SL5004	Procrit	1.56e-09	6.53e-12	178000	657	5.59e-07	6.00e-07
SL5004	Procrit	9.47e-10	5.06e-12	255000	1230	5.51e-07	6.00e-07
SL5004	Procrit	2.69e-10	4.05e-12	664000	7970	1.63e-06	1.60e-06
SL5004	Procrit	9.45e-10	1.11e-11	7.19e+05	7750	3.08e-06	3.10e-06
SL5004	Procrit	5.83e-10	6.04e-12	9.65e+05	8990	2.54e-06	2.50e-06
SL5004	Procrit	9.64e-10	3.95e-12	4.12e+05	1540	6.71e-07	7.00e-07
SL5004	Procrit	1.64e-09	2.46e-11	2.32e+05	3440	9.15e-07	9.00e-07
SL5004	Procrit	9.58e-10	1.89e-11	4.82e+05	9410	1.32e-06	1.30e-06
