# Supplementary material for: Modified aptamers as reagents to characterize recombinant human erythropoietin products
Source: Sci Rep. 2020 Oct 29;10:18593. doi: 10.1038/s41598-020-75713-2 (PMC7596557; doi:10.1038/s41598-020-75713-2)
Supplement: Supplementary file 3 — Supplementary Table 3. [file 41598_2020_75713_MOESM3_ESM.pdf]

Supplemental Table 3:

SOMAmer	Product	Measurement	P-Value	Adjusted P-value
SL5001	Procrit	KD	0.5658	0.7230
SL5001	Procrit	ka	0.9677	0.9892
SL5001	Procrit	kd	0.6588	0.7752
SL5001	BIS-1	KD	0.4386	0.6439
SL5001	BIS-1	ka	0.4855	0.6624
SL5001	BIS-1	kd	0.3865	0.6353
SL5001	BIS-2	KD	0.4286	0.6439
SL5001	BIS-2	ka	0.4266	0.6439
SL5001	BIS-2	kd	0.3223	0.5797
SL5001	BIS-3	KD	0.4833	0.6624
SL5001	BIS-3	ka	0.7882	0.8633
SL5001	BIS-3	kd	0.4032	0.6359
SL5001	NLP-1	KD	0.3188	0.5797
SL5001	NLP-1	ka	0.4040	0.6359
SL5001	NLP-1	kd	0.0073	0.0842
SL5001	NLP-2	KD	0.8905	0.9453
SL5001	NLP-2	ka	0.4445	0.6457
SL5001	NLP-2	kd	0.4561	0.6488
SL5001	NLP-3	KD	0.0071	0.0842
SL5001	NLP-3	ka	0.2053	0.5243
SL5001	NLP-3	kd	0.0171	0.1685
SL5001	NLP-4	KD	0.9549	0.9834
SL5001	NLP-4	ka	0.7599	0.8389
SL5001	NLP-4	kd	0.9864	0.9906
SL5001	NLP-5	KD	0.2996	0.5797
SL5001	NLP-5	ka	0.0937	0.3495
SL5001	NLP-5	kd	0.9800	0.9906
SL5001	NLP-6	KD	0.3366	0.5807
SL5001	NLP-6	ka	0.0915	0.3495
SL5001	NLP-6	kd	0.1721	0.4947
SL5001	NLP-7	KD	0.7395	0.8230
SL5001	NLP-7	ka	0.4514	0.6488
SL5001	NLP-7	kd	0.8280	0.8927
SL5001	NLP-8	KD	0.6401	0.7615
SL5001	NLP-8	ka	0.5018	0.6723
SL5001	NLP-8	kd	0.7056	0.8048
SL5002	Procrit	KD	0.2107	0.5243
SL5002	Procrit	ka	0.1933	0.5243
SL5002	Procrit	kd	0.1928	0.5243
SL5002	BIS-1	KD	0.5789	0.7292
SL5002	BIS-1	ka	0.4313	0.6439
SL5002	BIS-1	kd	0.2124	0.5243
SL5002	BIS-2	KD	0.2165	0.5243
SL5002	BIS-2	ka	0.2146	0.5243
SL5002	BIS-2	kd	0.1302	0.4277
SL5002	BIS-3	KD	NA	NA
SL5002	BIS-3	ka	NA	NA
SL5002	BIS-3	kd	NA	NA
SL5002	NLP-1	KD	0.1711	0.4947
SL5002	NLP-1	ka	0.3417	0.5821
SL5002	NLP-1	kd	0.0603	0.2774
SL5002	NLP-2	KD	0.0489	0.2774
SL5002	NLP-2	ka	0.2305	0.5378
SL5002	NLP-2	kd	0.0700	0.3018
SL5002	NLP-3	KD	0.0495	0.2774
SL5002	NLP-3	ka	0.2455	0.5378
SL5002	NLP-3	kd	0.0726	0.3036
SL5002	NLP-4	KD	NA	NA
SL5002	NLP-4	ka	NA	NA
SL5002	NLP-4	kd	NA	NA
SL5002	NLP-5	KD	0.1614	0.4843
SL5002	NLP-5	ka	0.0257	0.1968
SL5002	NLP-5	kd	0.0686	0.3018
SL5002	NLP-6	KD	0.3319	0.5797
SL5002	NLP-6	ka	0.9304	0.9727
SL5002	NLP-6	kd	0.2520	0.5434
SL5002	NLP-7	KD	0.9434	0.9789
SL5002	NLP-7	ka	0.5277	0.6909
SL5002	NLP-7	kd	0.7036	0.8048
SL5002	NLP-8	KD	0.8999	0.9480
SL5002	NLP-8	ka	0.2633	0.5579
SL5002	NLP-8	kd	0.9906	0.9906
SL5003	Procrit	KD	0.5650	0.7230
SL5003	Procrit	ka	0.6016	0.7360
SL5003	Procrit	kd	0.6009	0.7360
SL5003	BIS-1	KD	0.2766	0.5697
SL5003	BIS-1	ka	0.0491	0.2774
SL5003	BIS-1	kd	0.4896	0.6624
SL5003	BIS-2	KD	0.3312	0.5797
SL5003	BIS-2	ka	0.3250	0.5797
SL5003	BIS-2	kd	0.6826	0.7915
SL5003	BIS-3	KD	0.0984	0.3558
SL5003	BIS-3	ka	0.0057	0.0842
SL5003	BIS-3	kd	0.4040	0.6359
SL5003	NLP-1	KD	0.1568	0.4809
SL5003	NLP-1	ka	0.0052	0.0842
SL5003	NLP-1	kd	0.0112	0.1187
SL5003	NLP-2	KD	0.0216	0.1843
SL5003	NLP-2	ka	0.0311	0.2257
SL5003	NLP-2	kd	0.2430	0.5378
SL5003	NLP-3	KD	0.0020	0.0673
SL5003	NLP-3	ka	0.0044	0.0842
SL5003	NLP-3	kd	0.0227	0.1843
SL5003	NLP-4	KD	0.5307	0.6909
SL5003	NLP-4	ka	0.6629	0.7752
SL5003	NLP-4	kd	0.8019	0.8714
SL5003	NLP-5	KD	0.2830	0.5698
SL5003	NLP-5	ka	0.3867	0.6353
SL5003	NLP-5	kd	0.7275	0.8162
SL5003	NLP-6	KD	0.1031	0.3558
SL5003	NLP-6	ka	0.6080	0.7360
SL5003	NLP-6	kd	0.2849	0.5698
SL5003	NLP-7	KD	0.2668	0.5579
SL5003	NLP-7	ka	0.0898	0.3495
SL5003	NLP-7	kd	0.5247	0.6909
SL5003	NLP-8	KD	0.4101	0.6359
SL5003	NLP-8	ka	0.1016	0.3558
SL5003	NLP-8	kd	0.4348	0.6439
SL5004	Procrit	KD	0.3862	0.6353
SL5004	Procrit	ka	0.3237	0.5797
SL5004	Procrit	kd	0.0493	0.2774
SL5004	BIS-1	KD	0.2930	0.5776
SL5004	BIS-1	ka	0.6382	0.7615
SL5004	BIS-1	kd	0.2274	0.5378
SL5004	BIS-2	KD	0.8748	0.9358
SL5004	BIS-2	ka	0.1521	0.4769
SL5004	BIS-2	kd	0.0194	0.1787
SL5004	BIS-3	KD	0.2117	0.5243
SL5004	BIS-3	ka	0.3199	0.5797
SL5004	BIS-3	kd	0.4831	0.6624
SL5004	NLP-1	KD	0.6050	0.7360
SL5004	NLP-1	ka	0.1207	0.4063
SL5004	NLP-1	kd	0.0009	0.0599
SL5004	NLP-2	KD	0.0071	0.0842
SL5004	NLP-2	ka	0.0557	0.2774
SL5004	NLP-2	kd	0.1504	0.4769
SL5004	NLP-3	KD	0.0556	0.2774
SL5004	NLP-3	ka	0.0873	0.3495
SL5004	NLP-3	kd	0.0038	0.0842
SL5004	NLP-4	KD	0.4717	0.6624
SL5004	NLP-4	ka	0.0529	0.2774
SL5004	NLP-4	kd	0.0343	0.2364
SL5004	NLP-5	KD	0.0582	0.2774
SL5004	NLP-5	ka	0.0015	0.0673
SL5004	NLP-5	kd	0.0071	0.0842
SL5004	NLP-6	KD	0.2374	0.5378
SL5004	NLP-6	ka	0.5813	0.7292
SL5004	NLP-6	kd	0.0003	0.0397
SL5004	NLP-7	KD	0.7268	0.8162
SL5004	NLP-7	ka	0.2014	0.5243
SL5004	NLP-7	kd	0.0603	0.2774
SL5004	NLP-8	KD	0.4078	0.6359
SL5004	NLP-8	ka	0.2391	0.5378
SL5004	NLP-8	kd	0.3110	0.5797
